# Adverse Prognostic Significance of Activation‐Induced Cytidine Deaminase in Diffuse Large B‐Cell Lymphoma Patients Treated With R‐CHOP

**DOI:** 10.1002/cnr2.70557

**Published:** 2026-04-28

**Authors:** Mardiah Suci Hardianti, Nungki Anggorowati, Syahru Agung Setiawan, Miraz Radhea Bagaskoro, Johan Kurnianda, Kartika Widayati Taroeno‐Hariadi, Ibnu Purwanto, Susanna Hilda Hutajulu

**Affiliations:** ^1^ Division of Hematology and Medical‐Oncology, Department of Internal Medicine Faculty of Medicine, Public Health and Nursing, Universitas Gadjah Mada/Dr. Sardjito Hospital Yogyakarta Indonesia; ^2^ Department of Anatomical Pathology Faculty of Medicine, Public Health and Nursing, Universitas Gadjah Mada/Dr. Sardjito Hospital Yogyakarta Indonesia

**Keywords:** AID, DLBCL, overall survival, rituximab

## Abstract

**Background:**

A number of studies have demonstrated the important role of activation‐induced cytidine deaminase (AID) in the pathogenesis of diffuse large B‐cell lymphoma (DLBCL). AID has been relatively underexplored as a prognostic factor in DLBCL, and its role remains controversial.

**Aims:**

This study conducted a comprehensive analysis of the association between AID expression and clinical outcomes in 70 Indonesian patients with DLBCL treated with a standard CHOP ± rituximab regimen.

**Methods and Results:**

Immunostaining results showed that AID was expressed in 35.7% of DLBCL samples. Multivariate analysis indicated that positive AID expression was associated with shorter overall survival (OS) specifically among patients receiving a rituximab‐containing regimen, with a hazard ratio of 10.39 (95% CI: 1.93–55.96; *p* = 0.006).

**Conclusion:**

The significant prognostic value of AID in the DLBCL subgroup treated with rituximab—but not in the general DLBCL population or in those not receiving rituximab—underscores the potential relevance of AID expression in DLBCL management and treatment decisions.

## 1 Introduction

Activation‐induced cytidine deaminase (AID) has been implicated in the pathogenesis of lymphoma over the last two decades. It has been extensively explored both from the perspective of the molecular mechanism of the disease and from a clinical perspective. Initially, its role in lymphoma pathogenesis was attributed to its physiological function in the process of somatic hypermutation (SHM) and class switch recombination (CSR) of the Immunoglobulin (Ig) gene in the germinal center [1].

Previous studies have identified AID expression in various types of lymphoma, including follicular lymphoma (FL), Burkitt's lymphoma (BL), diffuse large B‐cell lymphoma (DLBCL), and lymphocyte‐originated tumors such as ALL, CLL, and MM [2, 3, 4]. Strong evidence suggests that AID plays a pivotal role in the pathogenesis of DLBCL [4, 5, 6]. In murine models, AID deficiency prevented germinal center‐derived lymphomagenesis in BCL6‐driven lymphomas but did not impact MYC‐driven, pre‐germinal center lymphomas, demonstrating that AID is specifically required for germinal center B‐cell‐derived lymphomas [7]. AID has been shown to promote class switch recombination, potentially leading to c‐MYC/IgH translocation in more aggressive DLBCL subtypes, particularly double‐hit lymphomas (DHL) [6]. Studies using chromatin immunoprecipitation sequencing analyses revealed that AID hotspots in both activated B cells and lymphoma cells in vitro were highly enriched for G4 elements, which facilitate genomic instability [8]. Frequent and elevated AID protein expression has been reported in HIV‐associated DLBCL relative to HIV‐negative DLBCL, independent of cell‐of‐origin, indicating a potential role for AID in lymphomagenesis in this context [9]. AID overexpression in DLBCL has also been linked to increased cytosine methylation heterogeneity, implicating AID‐mediated DNA demethylation and epigenetic diversification as contributors to lymphoma pathobiology [10].

However, the role of AID as a prognostic factor in DLBCL remains controversial and explored to a limited extent in the context of rituximab‐based immunotherapy. It has been reported that AID mRNA expression predicts an unfavorable outcome in DLBCL patients treated with conventional Cyclophosphamide‐Doxorubicin‐Vincristine‐Prednisone (CHOP) chemotherapy without rituximab therapy [11]. Recent evidence suggests that AID expression specifically associates with disease control and may influence treatment response in patients with IPI‐high scores [12]. The significance of AID in the context of rituximab‐containing regimens warrants further investigation, as rituximab addition to standard chemotherapy has substantially improved outcomes in DLBCL. Additionally, molecular biomarkers have been increasingly linked AID to lymphoma pathogenesis and treatment response. Studies across different populations and treatment modalities are necessary to clarify the prognostic role of AID [13].

In this study, we investigated AID protein expression in Indonesian DLBCL patients and its association with clinical outcomes following treatment with the standard CHOP ± rituximab regimen, with particular focus on the rituximab‐treated subgroup.

## 2 Materials and Methods

### 2.1 Patients

This was a retrospective cohort study conducted at Sardjito General Hospital, Yogyakarta, Indonesia. We extracted clinical data from medical records of DLBCL patients diagnosed and treated between January 2014 and December 2019. Data extraction was initiated on December 28th, 2019, after ethical clearance was obtained. Inclusion criteria were: (1) age ≥ 18 years; (2) biopsy‐confirmed diagnosis of DLBCL according to WHO classification; (3) treatment with standard chemotherapy regimen (rituximab‐CHOP [R‐CHOP] or CHOP alone); (4) adequate tissue specimens for immunohistochemistry analysis; and (5) follow‐up data available for at least 3 years or until death. Exclusion criteria included: (1) primary CNS lymphoma; (2) lymphomas with prior therapy; (3) incomplete clinical data; and (4) inadequate tissue quality for immunohistochemistry.

The follow‐up period was defined as 3 years from the time of initial diagnosis. Overall survival (OS) was defined as the time interval between the day of initial diagnosis and the day of death due to any cause. The joint ethics committee of the Faculty of Medicine, Public Health and Nursing, Universitas Gadjah Mada and Dr. Sardjito General Hospital approved this study on December 18, 2018 (KE/FK/1325/EC/2018), in accordance with the Declaration of Helsinki.

A total of 70 samples of DLBCL were collected from the pathological repository in the Department of Pathological Anatomy. The data from the medical record contained the patients' age, Ann Arbor stadium, extranodal involvement, eastern cooperative oncology Group (ECOG) performance status, lactate dehydrogenase (LDH) level, chemotherapy regimen, and overall survival status within 3 years of follow‐up. In previous studies, Hans' algorithm was used to determine the cell of origin (COO) by including CD10, BCL6, and MUM1, with germinal center B‐cells‐like subtype (GCBs) being CD10 + or BCL6 +/CD10–/MUM1 or IRF4– and non‐GCBs being CD10–/MUM1 or IRF4+ (BCL6 positive or negative) [14].

### 2.2 Immunohistochemistry Study

We cut formalin‐fixed paraffin‐embedded (FFPE) tissue blocks into four‐micrometer‐thick sections. Each section was adhered to a coated slide (Biogear Scientific, Coralville, Iowa, USA). We performed dehydration of the sections overnight at 45°C. Deparaffinization was performed by bathing sections in xylene (three times, 5 min each) followed by graded ethanol (100%, 95%, 70%, 50%; 3 min each) for rehydration. The antigen retrieval step was conducted in a Decloacking apparatus. Mouse anti‐AID monoclonal antibody was diluted to 1:50 for use as the primary antibody (Cat. No. 14‐5959‐82, Invitrogen, eBioscience, San Diego, California, USA). Primary antibody incubation was performed for 60 min at room temperature. Thereafter, anti‐mouse secondary antibodies (Cat. No. NB7535, Novus Biologicals, Colorado, USA) were stained with a dilution of 1:1000 for 30 min at room temperature. Hematoxylin was then used to counterstain the sample for 3 min. Upon completion of counterstaining, mounting was performed as a final immunostaining step.

Images were acquired at 400× magnification (high magnification, scale bar = 50 μm) and 100× magnification (low magnification, scale bar = 100 μm) using a Leica Microsystems microscope (Leica Microsystems, Wetzlar, Germany) and confirmed by a pathology expert. A single expert pathologist, blinded to clinical data, assessed AID expression and performed all tissue scoring to minimize observer bias. Reactive lymph node tissue samples were positive indicators. As a negative control, the primary antibody was omitted. Pathological evaluation was performed only on malignant B cells in the tumoral tissue. The observer counted a minimum of 100 malignant cells per case to ensure adequate sampling. Positive AID expression was defined as > 20% of malignant cells, as previously described [15].

### 2.3 Statistical Analysis

Chi‐square or Fisher's exact test was used for comparing proportions between groups. The Kaplan–Meier survival analysis was used to assess the OS distributions in relation to immunohistochemistry results of AID. Log‐rank test was used to compare survival differences among certain groups of patients. Univariate Cox proportional hazards regression analysis was performed to assess association between each variable and overall survival, yielding crude hazard ratios (HRs) with 95% confidence intervals (CIs). Subsequent multivariate Cox regression analysis was conducted to determine adjusted hazard ratios, accounting for potential confounding variables. Subgroup analyses were performed to evaluate whether AID's prognostic significance varied across clinically relevant groups of patients, including IPI risk categories, cell of origin, and treatment regimen. The statistical analysis was conducted using R (v4.2.3, R Foundation for Statistical Computing, Vienna, Austria). Statistical significance was defined as *p* < 0.05.

## 3 Results

### 3.1 Clinical Characteristics of AID Expression in DLBCL

Among 70 DLBCL samples analyzed, 25/70 (35.7%) were AID‐positive and 45/70 (64.3%) were AID‐negative in malignant cells. Rituximab‐containing regimen was administered to 32/70 (45.7%) patients, while 38/70 (54.3%) received CHOP‐alone. Within the 3‐year surveillance period, 48/70 (68.5%) patients survived.

Baseline clinical and pathological characteristics were compared between AID‐positive and AID‐negative groups (Table 1). There were no statistically significant differences between the two groups regarding age, gender, ECOG performance status, disease stage, extranodal involvement, LDH level, IPI score, cell of origin subtype, or treatment modality. In the CHOP‐alone group, 13 of 38 patients (34.2%) were AID‐positive, while in the R‐CHOP group, 12 of 32 patients (37.5%) were AID‐positive.

**TABLE 1:** Characteristics of DLBCL patients according to AID expression.

Parameter		AID negative	AID positive	*p*
*n*		45	25	
Age (median [IQR]), years		59.00 (50.00, 65.00)	60.00 (54.00, 65.00)	0.380
Gender (%)	Female	19 (42.2)	12 (48.0)	0.830
	Male	26 (57.8)	13 (52.0)	
Age group (%)	≤ 60 y.o	27 (60.0)	13 (52.0)	0.692
	> 60 y.o	18 (40.0)	12 (48.0)	
Ann‐arbor stage (%)	Stage I/II	29 (64.4)	21 (84.0)	0.144
	Stage III/IV	16 (35.6)	4 (16.0)	
Extranodal involvement (%)	No/single	40 (88.9)	24 (96.0)	0.410
	Multiple (≥ 2)	5 (11.1)	1 (4.0)	
ECOG PS (%)	0–2	40 (88.9)	24 (96.0)	0.410
	> 2	5 (11.1)	1 (4.0)	
LDH level (%)	Normal	36 (80.0)	19 (76.0)	0.931
	Elevated	9 (20.0)	6 (24.0)	
IPI risk score (%)	Low (0–1)	32 (71.1)	20 (80.0)	0.596
	High [2, 3, 4, 5]	13 (28.9)	5 (20.0)	
Cell of origin (%)	GCB	11 (24.4)	7 (28.0)	0.967
	Non‐GCB	34 (75.6)	18 (72.0)	
Treatment (%)	Without rituximab	25 (55.6)	13 (52.0)	0.971
	With rituximab	20 (44.4)	12 (48.0)	
Patients' status (%)	Alive	31 (68.9)	17 (68.0)	1.000
	Dead	14 (31.1)	8 (32.0)	

Abbreviations: CI, confidence interval; ECOG PS, eastern cooperative oncology group performance status; GCB, germinal center B‐cells; IPI, international prognostic Index; IQR, interquartile range; LDH, lactate dehydrogenase.

To assess whether rituximab treatment was associated with differences in cell of origin (COO) distribution, a supplementary analysis was performed (Table S1). Among the 70 cases evaluated, 18 were classified as germinal center B‐cell‐like (GCB) and 52 as non‐GCB. In the CHOP‐alone group, 13 of 18 (72.2%) were GCB, whereas in the rituximab‐treated group, 27 of 52 (51.9%) were non‐GCB. No statistically significant difference was observed in the proportion of GCB and non‐GCB subtypes between the two treatment groups (*p* = 0.102), suggesting that treatment allocation was relatively balanced across these biologic categories.

### 3.2 Immunostaining Expression of AID in DLBCL

**FIGURE 1:**
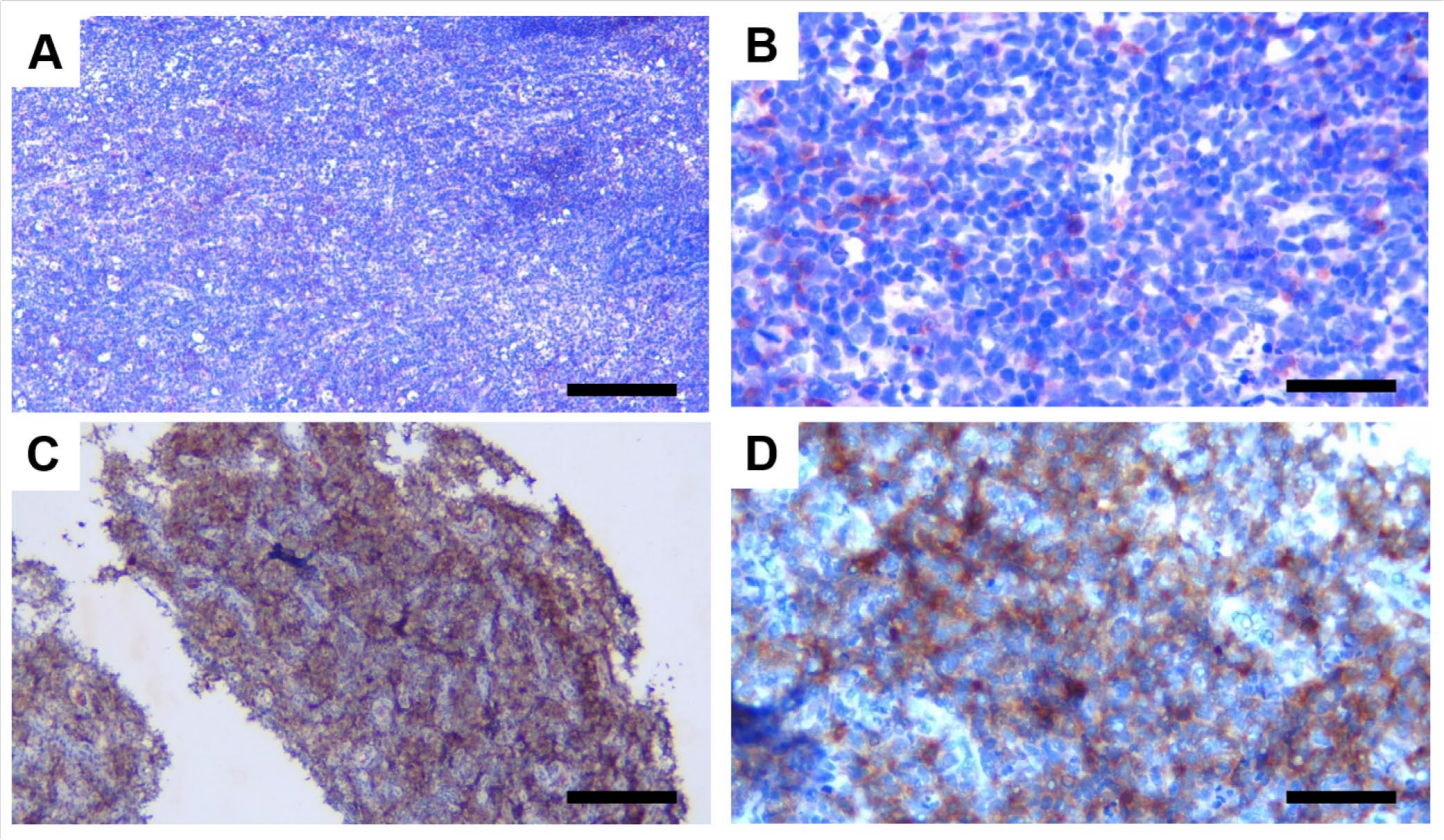
Representative images of immunostaining in tumor biopsy delineating AID‐positive and AID‐negative of DLBCL. Panels (A) and (C) presented low magnification views (100×) with a scale bar of 100 μm, illustrating AID‐negative (A) and AID‐positive (C) staining patterns in DLBCL tissues. Panels (B) and (D) showed corresponding high magnification views (400×) with a scale bar of 25 μm, highlighting detailed cellular features of AID‐negative (B) and AID‐positive (D) samples.

AID expression was predominantly localized in the cytoplasm of malignant B cells, with limited cases showing nuclear staining. Both dark and light zones of germinal center‐like structures within the tumor area expressed AID at varying intensities. Representative immunohistochemical images of AID‐positive and AID‐negative DLBCL cases are shown in Figure 1 (400× magnification for individual cell assessment, 100× magnification for tissue architecture overview).

### 3.3 Prognostic Relevance of AID Expression in DLBCL

**FIGURE 2:**
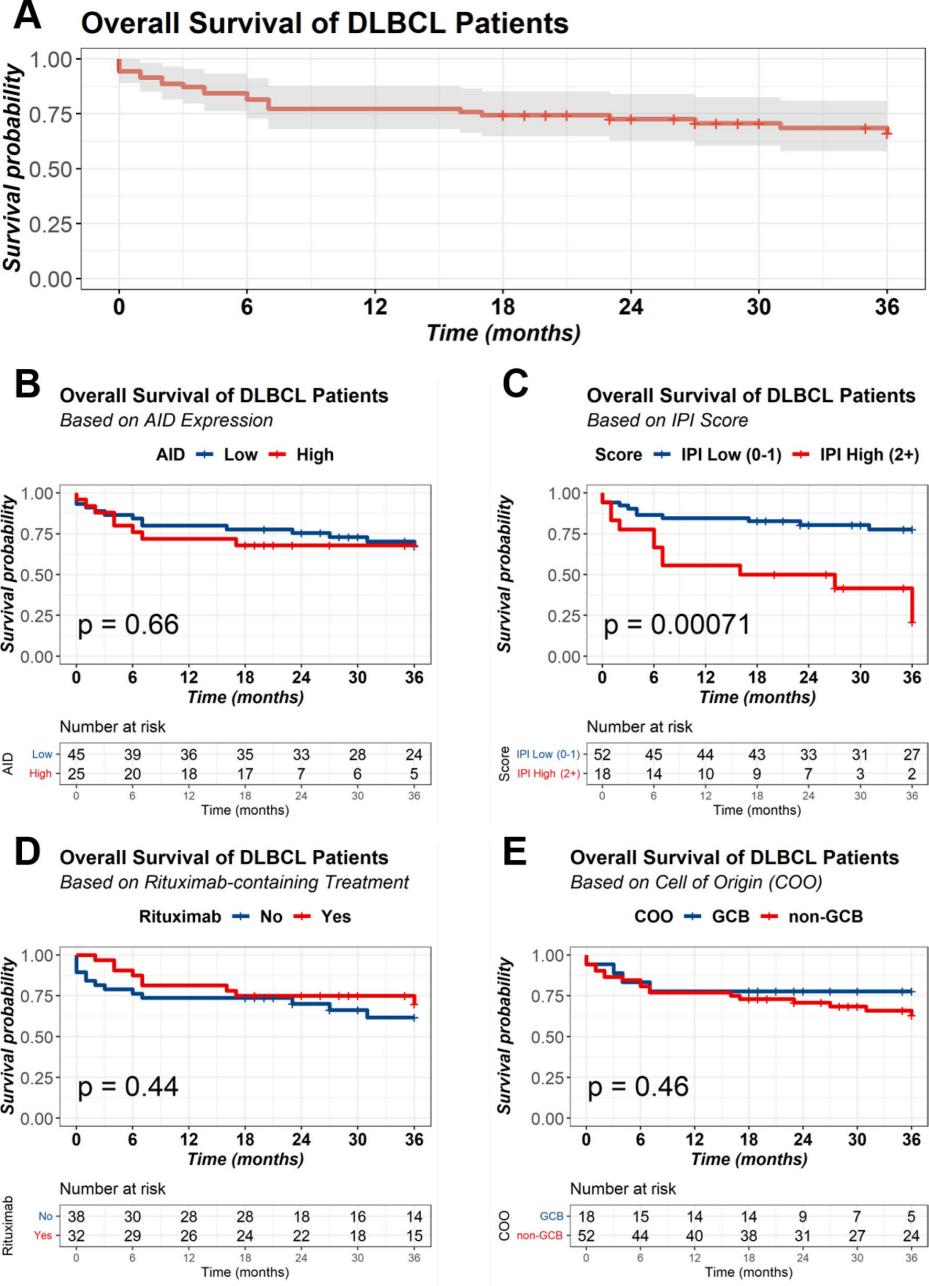
Kaplan–Meier curve of overall survival (OS) in DLBCL Patients. OS in whole cohort (A) and stratified according to the AID expression (B), IPI score (C), rituximab‐containing regimen (D), cell of origin (COO) subtype (E).

Within the 3‐year surveillance period, median OS was 28.5 months (Figure 2A). In the overall cohort, AID‐positive patients had a mean OS of 26 months compared with 28 months in AID‐negative patients, though this difference was not statistically significant (*p* = 0.66) (Figure 2B). Patients with high IPI score (≥ 2) had significantly worse OS than those with (< 2) (*p* = 0.00071) as represented in (Figure 2C). Treatment outcomes were not significantly different between the groups of Rituximab‐containing regimen and those receiving CHOP alone (Figure 2D). There was no difference in the OS when the patients were stratified according to the COO of the disease (*p* = 0.46), although non‐GCB tended to show worse outcome (Figure 2E).

Univariate and multivariate Cox‐regression analyses were performed to assess the relationship between potential prognostic factors and OS in the entire cohort (Table 2). Compared with AID‐negative patients, those with positive AID expression had a crude HR of 1.22 (95% CI: 0.51–2.93, *p* = 0.661), which was not statistically significant. High IPI risk score (≥ 2) remained an independent prognostic factor with an adjusted HR of 4.18 (95% CI: 1.76–9.93, *p* = 0.001). Rituximab addition to CHOP showed a protective effect with an adjusted HR of 0.62 (95% CI: 0.26–1.49, *p* = 0.288), though this did not reach statistical significance. Non‐GCB derivation had an adjusted HR of 1.76 (95% CI: 0.58–5.35, *p* = 0.322) compared to the GCB subtype.

**TABLE 2:** Univariate and multivariate Cox‐regression analysis of prognostic factors in DLBCL overall survival.

Parameter (*n* = 70)		*N* (%)	Crude HR (95% CI, *p*)	Adjusted HR (95% CI, *p*)
AID expression	Negative	45 (64.3)	—	—
	Positive	25 (35.7)	1.22 (0.51–2.93, *p* = 0.661)	1.44 (0.59–3.49, *p* = 0.421)
IPI risk score	Low (0–1)	52 (74.3)	—	—
	High (2–5)	18 (25.7)	3.92 (1.67–9.16, *p* = 0.002)	4.18 (1.76–9.93, *p* = 0.001)
Rituximab‐based treatment	No	38 (54.3)	—	—
	Yes	32 (45.7)	0.71 (0.30–1.67, *p* = 0.434)	0.62 (0.26–1.49, *p* = 0.288)
Cell of origin (COO) subtype	GCB	18 (25.7)	—	—
	Non‐GCB	52 (74.3)	1.50 (0.51–4.45, *p* = 0.462)	1.76 (0.58–5.35, *p* = 0.322)

Abbreviations: GCB, germinal center B‐cells; HR, hazard ratio; IPI, international prognostic index.

**FIGURE 3:**
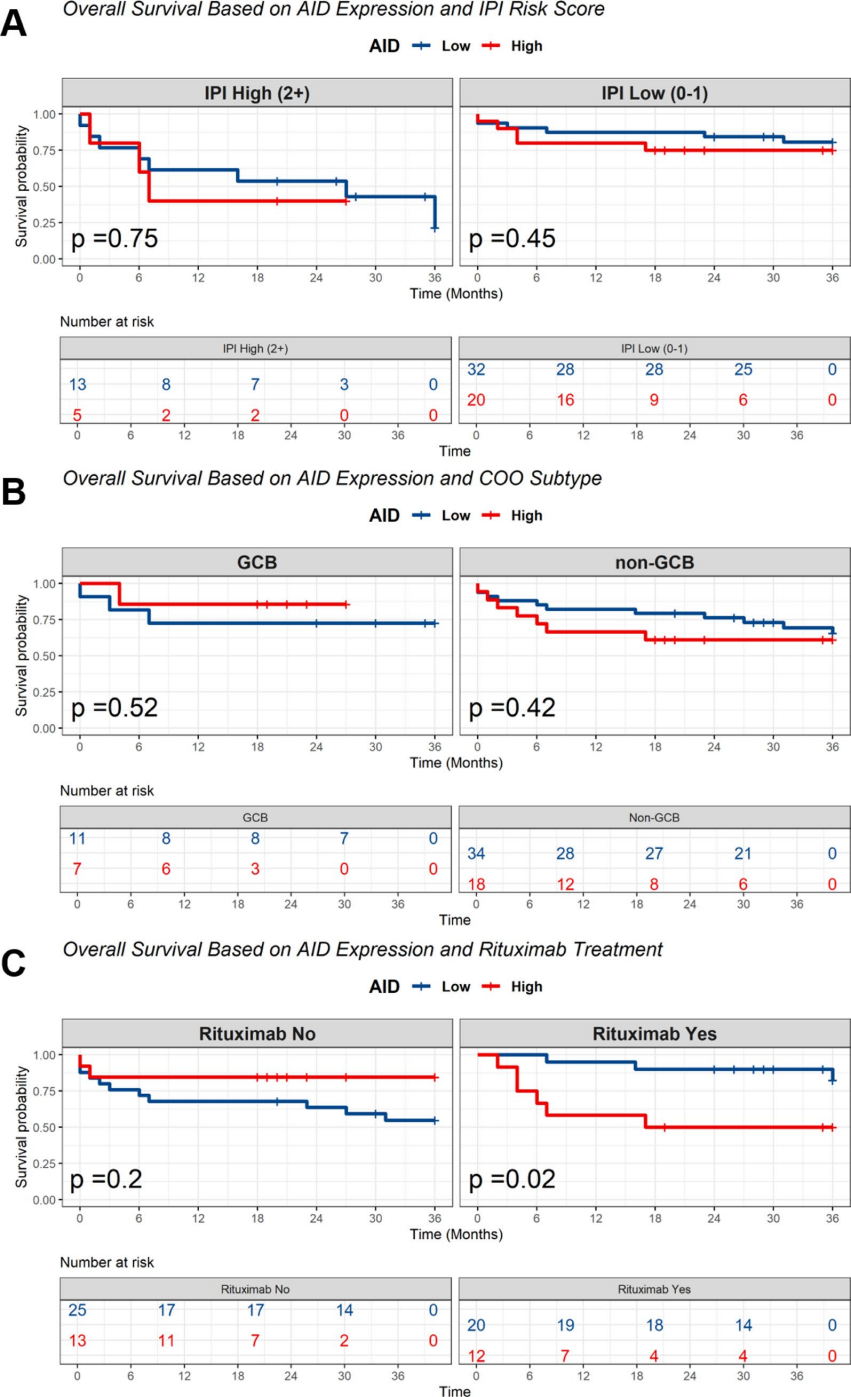
Subgroup analysis of AID expression in DLBCL patients. OS was stratified based on IPI risk score (A), cell of origin (COO) subtype (B), and rituximab‐added treatment (C).

Subgroup analysis of AID expression according to IPI score, COO subtype and Rituximab‐containing treatment was then performed (Figure 3). In this part, the expression of AID did not significantly associate with DLBCL overall survival in different subgroups of IPI risk score (high and low score) and COO subtype (GCB and non‐GCB) (Figure 3A,B). AID positive among non‐GCB subtype of DLBCL patients tended to have worse outcome than AID negative although the difference was not statistically significant (*p* = 0.42) (Figure 3B). Interestingly, positive expression of AID was associated with significantly poor survival among DLBCL patients who received rituximab‐containing treatment (*p* = 0.02) (Figure 3C).

**FIGURE 4:**
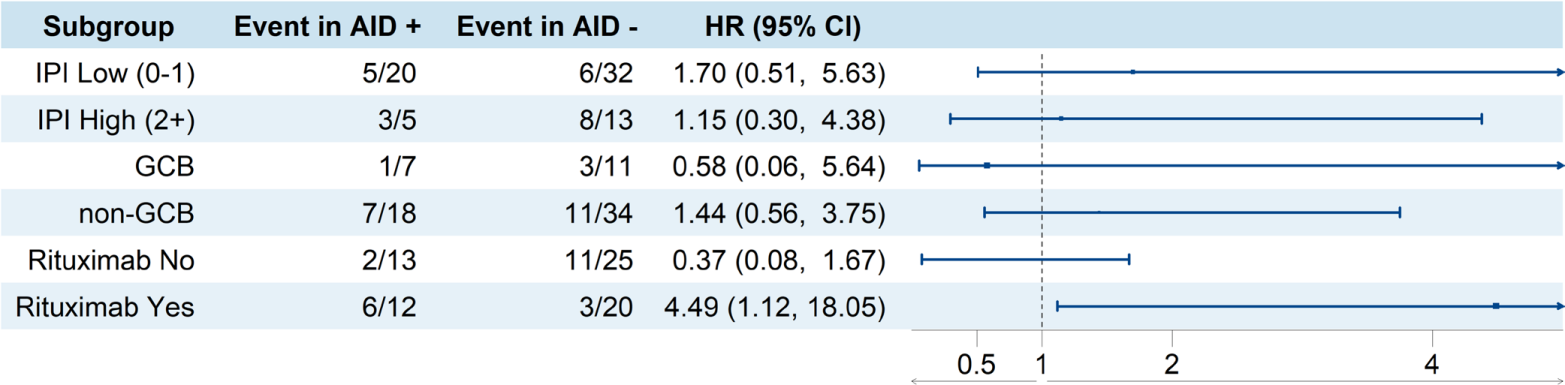
Hazard ratio of AID positive for DLBCL subgroups defined by IPI score, cells of origin (COO), and rituximab‐containing treatment. Forest plot demonstrated that AID positive status shows adverse prognostic significance specifically in the rituximab‐containing treatment subgroup.

Forest plot analysis was used to visualize the impact of AID expression on OS across different DLBCL subgroups (Figure 4). Previously, in overall DLBCL patients, AID did not show a significant association to affect DLBCL survival (Figure 2B). However, among subgroups of DLBCL patients who received rituximab‐containing regimens, positive AID expression was significantly associated with poorer survival (HR 4.80; 95% CI: 1.19–19.39) (Figure 4). Thus, it appears that AID expression has prognostic value in the case of DLBCL patients receiving rituximab‐containing mainstay therapy.

To identify independent prognostic factors specifically in rituximab‐treated DLBCL patients (*N* = 32), multivariate Cox‐regression analysis was performed (Table 3). Both AID expression and IPI risk score emerged as independent prognostic factors for OS in this subgroup, with adjusted HRs of 10.39 (95% CI: 1.93–55.96, *p* = 0.006) and 9.89 (95% CI: 2.13–46.03, *p* = 0.003), respectively. Cell of origin subtype did not reach statistical significance as an independent prognostic factor in this rituximab‐treated subgroup (adjusted HR = 0.53, *p* = 0.590). Given the limited number of rituximab‐treated patients (*n* = 32) and events, estimates of effect size showed considerable variability, as reflected by the wide confidence intervals, and should therefore be interpreted with caution.

**TABLE 3:** Univariate and multivariate Cox‐regression analysis of prognostic factors in the overall survival of DLBCL patients receiving rituximab‐containing regimen.

Parameter (*n* = 32)		*N* (%)	Crude HR (95% CI, *p*)	Adjusted HR (95% CI, *p*)
AID expression	Negative	20 (62.5)	—	—
	Positive	12 (37.5)	4.80 (1.19–19.39, *p* = 0.028)	10.39 (1.93–55.96, *p* = 0.006)
IPI risk score	Low (0–1)	24 (75.0)	—	—
	High (2–5)	8 (25.0)	4.84 (1.25–18.72, *p* = 0.022)	9.89 (2.13–46.03, *p* = 0.003)
Cell of origin subtype	GCB	5 (15.6)	—	—
	Non‐GCB	27 (84.4)	1.40 (0.18–11.23, *p* = 0.749)	0.53 (0.05–5.30, *p* = 0.590)

Abbreviations: GCB, germinal center B‐cells; HR, hazard ratio; IPI, international prognostic index.

## 4 Discussion

In this cohort of 70 Indonesian DLBCL patients, AID protein expression was detected in 35.7% (25/70) of cases by immunohistochemistry, comparable to previous reports showing AID positivity in approximately 40%–45% of DLBCL cases [11, 12]. No significant differences were observed in baseline clinical characteristics, laboratory parameters, or cell‐of‐origin subtypes according to AID expression, consistent with prior findings that AID expression from the lymph nodes did not distinguish baseline features in DLBCL [12].

AID has been extensively implicated in lymphomagenesis through its role in somatic hypermutation and class‐switch recombination, processes that can induce chromosomal aberrations such as MYC/IgH translocations [4, 16]. Experimental models demonstrate that AID is essential for germinal center–derived lymphomagenesis, as AID deficiency abrogates lymphoma development in BCL6‐driven models but not in pre‐germinal center MYC‐driven lymphomas [7]. Genome‐wide chromatin analyses further show that AID preferentially targets G‐quadruplex‐rich regions, predisposing to off‐target mutations and genomic instability [8].

Molecular subtyping based on gene expression profiling has revealed that GCB and Activated B‐cell‐like (ABC) DLBCL represent biologically and clinically distinct entities [17] Moreover, recent studies highlight the context‐dependent effects of AID expression [18]. Although AID is traditionally associated with germinal center B‐cell biology, its expression spans both GCB and ABC DLBCL subtypes [17, 18]. In GCB DLBCL, AID activity may facilitate BCL2 and/or MYC translocations, characteristic of double‐hit lymphomas [6]. In contrast, in ABC DLBCL, which is driven by chronic B‐cell receptor and Toll‐like receptor signaling, AID‐induced mutations may further disrupt BCR signaling components or promote alternative NF‐κB–dependent survival pathways [19, 20]. Recurrent MYD88, CD79B, and CARD11 mutations converge on sustained NF‐κB activation, defining a biologically distinct ABC‐type subgroup [19, 20].

A key finding of this study is the treatment‐dependent prognostic relevance of AID expression. AID protein expression did not predict survival in the overall cohort but emerged as a strong independent adverse prognostic factor in patients receiving rituximab‐containing regimens. This effect was not observed in patients treated with CHOP alone, suggesting a specific interaction between AID‐related biology and rituximab response. Although the IPI remained a dominant prognostic factor, AID retained prognostic significance after multivariable adjustment in the rituximab‐treated subgroup, indicating an effect beyond established clinical risk parameters.

Rituximab exerts its antitumor effects through antibody‐dependent cellular cytotoxicity, complement‐dependent cytotoxicity, and direct apoptosis induction [21], and has also been shown to modulate BCR signaling via BLNK‐ and BTK‐dependent pathways [22]. In this context, AID‐mediated off‐target mutations affecting BCR signaling components or GC‐associated DNA lesions may impair rituximab‐induced apoptosis [4, 6, 23]. This mechanism may be particularly relevant in non‐GCB or ABC DLBCL, where constitutive BCR signaling predominates, potentially explaining the trend toward poorer outcomes in AID‐positive cases.

In the present cohort, non‐GCB cases were more frequent in the rituximab‐treated group than in the CHOP‐alone group, although this difference was not statistically significant (Table S1). This finding suggests that the adverse outcome observed among AID‐positive patients treated with rituximab is unlikely to be solely explained by an imbalance in cell‐of‐origin subtype. However, given the limited sample size, inferences regarding the interaction between AID expression, cell of origin, and treatment modality should be interpreted cautiously. Notably, the prognostic impact of AID was observed only in patients receiving rituximab‐containing regimens and not in the overall cohort or CHOP‐alone subgroup, indicating a potential treatment‐context–dependent association.

Although this study assessed AID protein expression rather than underlying genetic alterations, the findings can be discussed in relation to established DLBCL biology. AID has been implicated in off‐target genomic instability, including aberrations involving BCL2 and MYC in GCB DLBCL, which are known to confer poor prognosis in R‐CHOP–treated patients [23, 24]. Importantly, the present cohort predominantly comprised non‐GCB cases within the rituximab‐treated subgroup, suggesting that the adverse association of AID expression observed here may not be restricted to germinal center–derived translocation events. Instead, AID expression may reflect broader genomic or signaling perturbations relevant across DLBCL subtypes [23].

Previous studies have reported inconsistent results regarding the prognostic significance of AID in DLBCL, particularly across different treatment eras [11, 12]. While AID expression has been associated with inferior outcomes in CHOP‐treated [11] or high‐risk cohorts [12], the current findings suggest that its prognostic relevance may vary depending on treatment context. These observations should be considered hypothesis‐generating, and larger studies with sufficient power to formally assess treatment–biomarker interactions are needed to clarify whether AID expression modulates response to rituximab‐containing therapy or represents a surrogate marker of aggressive disease biology.

Although the present findings do not establish AID expression as a predictive biomarker for treatment selection, they raise the possibility that AID‐positive DLBCL represents a biologically distinct subgroup that may respond differently to rituximab‐containing therapy. As such, AID expression may warrant further investigation as a stratification variable in future studies rather than as a basis for immediate treatment modification. Beyond its prognostic association, AID has also emerged as a potential therapeutic target in DLBCL, particularly in relapsed or refractory disease. One proposed mechanism involves dysregulation of Fanconi anemia complementation group A (FANCA), which can be downregulated through active demethylation in the presence of AID and TET2, thereby sensitizing lymphoma cells to apoptosis. Preclinical studies have shown that inhibition of AID and TET2, including in combination with bortezomib, can induce apoptosis in DLBCL cells [25]. Additionally, another study group developed a direct AID inhibitor using ultrasound irradiation to generate reactive oxygen species (ROS), which increased apoptosis rates in DLBCL cells [26]. Hence, several strategies warrant investigation: (1) Combination of ibrutinib, a Bruton tyrosine kinase (BTK) inhibitor, with R‐CHOP for AID‐positive DLBCL, particularly in non‐GCB cases, as BTK inhibition has shown activity in BCR pathway‐dependent lymphomas [27, 28, 29]; (2) Direct targeting of AID in relapsed/refractory cases, using agents that block AID activity or induce apoptosis through alternative mechanisms [6, 26].

Strengths of this study include the comprehensive analysis of AID protein expression and clinical outcomes in a defined population‐based cohort. The multivariate statistical approach controlled for known prognostic factors (IPI, COO) to establish AID as an independent predictor. However, several limitations should be acknowledged. The overall sample size was modest, and the rituximab‐treated subgroup included only 32 patients with a limited number of events. Although the multivariable analysis demonstrated statistical significance, the small event count relative to the number of covariates may have introduced model instability and potential overfitting, as reflected by a high hazard ratio estimate (approximately 10) along with a wide confidence interval. These findings should therefore be interpreted with caution and warrant validation in larger, independent cohorts. Second, this study was conducted in a single institution in Indonesia, and findings may not be generalizable to other populations. Third, this study did not characterize mutations in key B‐cell receptor pathway genes (such as MYD88, CD79A/B, CARD11) or integrate AID expression with the mutation‐defined genetic subgroups now recognized in DLBCL, which might have provided mechanistic insight into differential rituximab responses [19, 20]. Fourth, tumor microenvironmental factors and immune cell composition were not evaluated, whereas recent data indicate that T‐cell– and macrophage‐based microenvironment signatures, particularly checkpoint‐positive T cells, can significantly influence response and survival in patients treated with rituximab‐containing regimens [30]. Finally, the 3‐year follow‐up period may not have captured long‐term outcomes, particularly for patients achieving prolonged remissions.

This study has provided valuable insights into the potential role of AID expression in guiding DLBCL management, particularly in the context of rituximab‐based immunotherapy. AID protein expression emerged as an independent adverse prognostic factor specifically for DLBCL patients receiving rituximab‐containing regimens, suggesting that treatment‐specific biomarker assessment may improve patient risk stratification and guide therapeutic decisions. Larger prospective, multicenter studies with balanced AID‐positive and AID‐negative cohorts are essential to validate these findings and investigate the mechanisms by which AID influences rituximab response, ultimately enabling the development of more targeted and effective therapies for AID‐positive DLBCL.

## Author Contributions

**Mardiah Suci Hardianti:** conceptualization, funding acquisition, formal analysis, methodology, project administration, writing – review and editing, writing – original draft. **Nungki Anggorowati:** investigation, visualization, software, writing – review and editing. **Syahru Agung Setiawan:** data curation, formal analysis, investigation, validation, writing – original draft. **Miraz Radhea Bagaskoro:** data curation, formal analysis, validation, visualization, writing – original draft. **Johan Kurnianda:** writing – review and editing, formal analysis, validation. **Kartika Widayati Taroeno‐Hariadi:** formal analysis, validation, writing – original draft. **Ibnu Purwanto:** formal analysis, validation, writing – review and editing. **Susanna Hilda Hutajulu:** formal analysis, validation, writing – original draft.

## Funding

This study was fully supported by PDUPT Grant of Indonesian Ministry of Research and Technology/National Agency for Research and Innovation 2020 (No. 2758/UN1.DITLIT/DIT‐LIT/PT/2020).

## Ethics Statement

The study was approved by The Ethical Committee of Faculty of Medicine, Universitas Gadjah Mada (approval no: KE/FK/1325/EC/2018). Tissue samples from patients with DLBCL tumors were obtained in compliance with the recommendations of the Declaration of Helsinki for Biomedical Research. Informed consent was obtained from subjects involved in the study.

## Conflicts of Interest

The authors declare no conflicts of interest.

## Supporting information


**Table S1:** Distribution of cell of treatment modality by cell of origin subtype.

## Data Availability

The data that support the findings of this study are available on request from the corresponding author. The data are not publicly available due to privacy or ethical restrictions.
