# Supplementary material for: Adverse Prognostic Significance of Activation‐Induced Cytidine Deaminase in Diffuse Large B‐Cell Lymphoma Patients Treated With R‐CHOP
Source: Cancer Rep (Hoboken). 2026 Apr 28;9(5):e70557. doi: 10.1002/cnr2.70557 (PMC13124646; doi:10.1002/cnr2.70557)
Supplement: Supplementary file 1 — Table S1: Distribution of cell of treatment modality by cell of origin subtype. [file CNR2-9-e70557-s001.docx]

**Table S1. Distribution of Cell of Treatment Modality by Cell of Origin Subtype**

| **Parameter** |  | **GCB** | **non-GCB** | **p** |
| --- | --- | --- | --- | --- |
| n |  | 18 | 52 |  |
| Rituximab-based treatment (%) | No | 13 (72.2) | 25 (48.1) | 0.134 |
|  | Yes | 5 (27.8) | 27 (51.9) |  |

GCB: Germinal center B-cells
